# Effect of conventional and household water treatment technologies on the removal of pesticide residues in drinking water, Jimma town, Southwestern, Ethiopia

**DOI:** 10.1371/journal.pone.0288086

**Published:** 2023-07-19

**Authors:** Temima Jemal, Higemengist Astatke, Amare Terfe, Seblework Mekonen

**Affiliations:** 1 Department of Environmental Health Science and Technology, Institute of Health, Jimma University, Jimma, Ethiopia; 2 Department of Environmental Health Science, College of Medicine and Health Sciences, Arba Minch University, Arba Minch, Ethiopia; 3 Ethiopian Institute of Water Resources, Water and Health, Addis Ababa University, Addis Ababa, Ethiopia; Fisheries College and Research Institute, INDIA

## Abstract

Water resources have been contaminated by pesticides due to the different activities of human beings. Different studies documented that advanced water treatment systems can eliminate pesticides while conventional and household treatment technologies are not well studied. The main aim of the present study is to determine the effect of conventional and household water treatment technologies on the removal of pesticide residue in drinking water. Water samples were collected from the Gibe River (intake point), from each treatment process, and from the distribution system. To determine the effect of the household water treatment process (solar disinfection and boiling), pesticides were spiked into distilled water and then passed through solar disinfection (SODIS) and boiling. The extraction of samples was conducted by following a low-density-based dispersive liquid-liquid microextraction procedure. The result of the study revealed that almost all studied pesticides except o´p-DDT were detected in water samples. Most pesticides that were detected in water samples from our study areas exceeded the maximum residue limits (MRLs), except for p,p’-DDE. The percent reduction of pesticide residue after post-chlorination by conventional water treatment ranges from 11.7% (from 70.83 μg/L to 62.54 μg/L) for p´p-DDD to 97.29% (5510.1μg/L to 149.5μg/L) for Dimethachlor, and the percent reduction of pesticide residue by SODIS and boiling ranges from 2.31% (o´p-DDT) to 54.45% (Cypermethrin) and 27.13% (γ-Chlordane) to 38.9% (p´p-DDE) respectively. This indicates that treatment technologies are important for the reduction of pesticides in water. Since studied pesticides are persistent and the resides were exceed MRL (have a health impact), monitoring of pesticides in treatment plant units is necessary and treatment technology improvement is important to allow further removal of pesticides.

## Introduction

Various types of pesticides are widely used in Ethiopia due to agricultural intensification. Even banned or severely restricted pesticides in other countries like Dichlorodiphenyltrichloroethane (DDT) and others are used illegally. Moreover, much misuse (abuse and overuse) of pesticides is practiced by Ethiopian farmers, particularly when storing, mixing (dosage), and applying them, and disposing of empty container [1]. In Ethiopia, pesticides are readily available at wholesale stores (importers), the farmers’ union and pesticides retailers with different chemical composition (organochlorines, organophosphates, pyrethroids and carbamates) most of the farmers (85%) get their pesticide supplies from private small shops[1,2]. Besides their intensive use, pesticides may leach from the irrigation of plants into the ground and surface water sources [3]. Moreover, only 0.1% of the pesticides reach the target during the application, while the remaining 99.9% have the potential to move to different environmental matrices, including surface and groundwater sources [4]. Farmyard runoff, sewer overflows, and accidental spills can contribute to the contamination of water sources especially rivers [5]. Pesticide residues were found in drinking water sources around the world, including Ethiopia [3,6–8]. Profenofos was detected at the highest concentration in the Bulbula River and in tap water at the Batu Drinking Water Supply in Ethiopia [7]. In the other study, Diazinon 2, 4-D, Malathion, Fenpropimorph, and Pirimiphos methyl were detected in water samples collected from different drinking water sources at Jimma Zone, and the study concluded that there was a decline in residue concentration from the source (river) to tap water except for Diazinon [8]. Consuming such polluted water may result in health problems for humans. Acute exposure to pesticides can lead to serious illness or death. Chronic exposure can impair the function of the endocrine, nervous, renal, immune, reproductive, respiratory, and cardiovascular systems[9,10]. According to a study done by [8], the pesticide residues detected in drinking water pose no acute health risk to consumers. However, chronic risks to human health were observed from exposure to some pesticides in the Jimma and Addis Ababa populations. Considering the toxicity of pesticides and their metabolites, the removal of pesticides from contaminated aquatic systems is necessary. According to different studies, advanced water treatment technologies, such as reverse osmosis, ozonation, advanced oxidation processes, adsorption, and nanofiltration, are effective in removing pesticide residues from water [11–17]. Despite this, advanced water treatment technologies are expensive, especially for countries like Ethiopia; however, conventional and household water treatment technologies can be an alternative. Even if the main purpose of conventional water treatment technologies is to remove microorganisms, and reduce turbidity and some chemicals. Very few studies in other countries like Malaysia and Iran, showed that conventional water treatment technology has an impact on reducing the levels of pesticides in water [3,6]. However, data were scarce about the effect of household water treatment technologies. In most developing countries, such as Ethiopia, there is a lack of studies conducted on the effect of both conventional and household water treatment processes on pesticide residues. The studied pesticides and the site for samples collection were slightly differ from the previous study. Therefore, the main aim of the present study is to determine the level of pesticides from sources up to the point of use, and the effect of conventional and household treatment technologies on the removal of pesticide residues.

## Materials and methods

### Description of the study area

**Fig 1:**
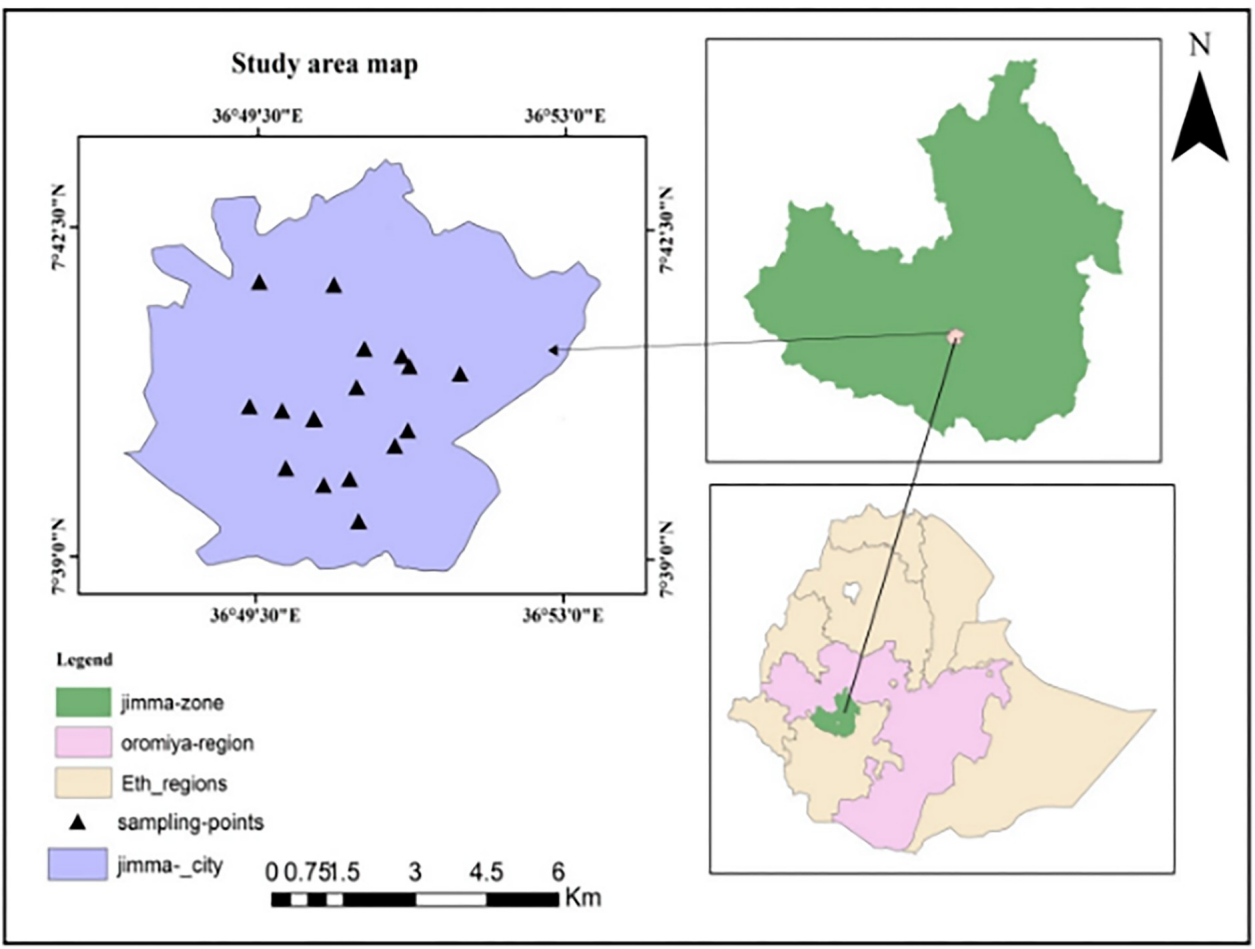
Map of study area in Jimma town, southwestern Ethiopia.

The study was conducted in Jimma, southwestern Ethiopia. Jimma is the largest city in southwestern Ethiopia (Fig 1). It has a latitude and longitude of (7° 40′ N, 36° 50′ E) and is located 350 km southwest of Addis Ababa at an average altitude of 1780 m above sea level. One of the sampling areas was Jimma Town’s drinking water treatment plant. The city uses a conventional water treatment system to purify water. The Gilgel Gibe River is the source of raw water for the Jimma Town water treatment plant. The river is bordered by wetlands, which harbor a high diversity of foraging and breeding birds. The discharge of untreated domestic, industrial, and intuitional wastewater, the disposal of solid waste, drainage, farming, clay mining, removal of riparian vegetation, and intensive livestock grazing threaten these valuable freshwater ecosystems [18]. During the observation there was agricultural activities around the river. The treatment plant has an intake structure with four low-lift pumps, and the aerator structure is a concrete cascade. It consists of treatment processes such as aeration, coagulation, flocculation units, sedimentation, filtration, and chlorination structures. The final treated water is lifted via high-lift pumps to a reservoir for distribution to the town. It served the town for about 20 years and was renovated in 2014 [19].

### Sampling of water

Purposive sampling was used in this study. Water samples were collected using grab sampling into 1 L of bottles, which were rinsed with ultrapure water, and then the bottles were flushed three times with the water to be sampled. After collection, the samples were labeled and transported to the Jimma University Environmental Health Laboratory, and stored at 4°C until further analysis. Before the extraction process, all water samples were filtered using Whatman filter paper with a pore size of 8μm to remove any particulate matter.

Three replicate water samples were collected from each treatment process to assess the fate of pesticide residues during conventional water treatment. The samples were collected from the raw water source (Gibe River), aeration effluent, coagulation effluent, flocculation effluent, sedimentation basin effluent, filtration effluent, and post-chlorination (disinfection) effluent. In total, 18 water samples were collected from the conventional water treatment plant in Jimma, southwestern Ethiopia.

To determine the concentration of pesticide residues in the water distribution system of Jimma town, water samples were collected from five reservoirs (tanks), and from each reservoir, three samples were collected. A total of 15 samples were collected, which were located in the treatment plant compound, Ginjo, Jimma University Medical Center, Abajifar, and Jiren. Andten water samples were collected from the community tap. The water samples taken from treatment plant, reservoirs and community tap were from July -August/2021.

### Effect of water treatment technologies on pesticide residues

The effect of water treatment technologies on pesticide residues was determined by calculating the percent reduction and processing factor.

Pesticide reduction at each water-treatment process was calculated using equation [3].

$$Percent\;reduction=\frac{concentration\;of\;pesticide\;in\;the\;raw\;water-concentration\;of\;pesticide\;under\;treatment\;x}{Concentration\;of\;pesticide\;in\;the\;raw\;water}*100$$ Eq (1)

Where x is the treatment process (aeration, coagulation and flocculation, sedimentation, filtration, post-chlorination SODIS, and boiling),

ORPercentreduction=(1−PF)*100

A percent reduction is expressed as a percentile. The positive sign of the present reduction indicated the reduction factor, whereas the negative sign of the percent reduction indicated the concentration factor.

Processing factor (PF) was calculated as the ratio between the pesticide concentrations in the processed water sample (μg/L) and the pesticide concentration in the unprocessed water sample (μg/L). PF < 1 indicated that there is a reduction of pesticides by the processing (reduction factor), whereas PF > 1 indicated no reduction by the processing (PF > 1 = concentration factor) [20].


$$Processing\;factor\;(PF)=\frac{concentration\;of\;processed\;sample}{concentration\;of\;pesticide\;in\;unprocessed(raw)\;sample}$$ Eq (2)

### Experimental procedure for Solar Disinfection (SODIS) and Boiling as household water treatment methods

SODIS and boiling (commonly practiced by the community) were selected as household treatment methods to see if they affected the concentration of pesticides in drinking water. This is because other household treatment mechanisms (filtration, sedimentation, and chlorination) were investigated under the conventional treatment process and there was a budget constraint to investigate all household water treatment methods. A common treatment process was used for both treatments. A 1ppm concentration of each pesticide was spiked into distilled water to assess the effect of SODIS and boiling on pesticide levels in the water. The SODIS intervention was designed according to the Swiss Federal Institute of Aquatic Science and Technology‘s published guideline [21] as follows:

✓ A 1 liter PET bottle was filled three-fourths with sampled water, thoroughly shaken to increase dissolved oxygen, fully filled, covered with an airtight lid, and placed horizontally on a reflective corrugated iron sheet. The bottle was exposed to the sun from morning until evening for at least 6 hours.
✓ For boiling, 1 liter of water was brought to a rolling point for 1 minute [22]

After treatment of spiked distilled water by SODIS and boiling, the extraction procedure was followed using low-density -based dispersive liquid-liquid micro-extraction.

### Chemicals and reagents

All chemicals and reagents were analytical and HPLC grade. n-hexane (99%purity), methanol (99% purity), and acetone (99.9%purity) were obtained from Biochem Pharm, France, and sodium chloride was used to improve the extraction of analytes due to the salting-out effect. N-hexane was used as an extraction solvent. As a dispersant solvent, acetone was used because it is partially miscible with water. High-purity analytical pesticide reference standards (98%) such as o´p-DDT, p´p-DDT, p´p-DDD, p´p-DDE, lindane, heptachlor, dimethachlor, γ-Chlordane, cypermethrin, and deltamethrin were used as an analyte for this study. Those pesticides were selected based on the availability of their standards and the others pesticides were investigated by other scholars [8,23]. Pesticides were obtained from Sigma Aldrich (St. Louis, United States of America).

### Preparation of standard solutions

Stock solutions of 1000 mg/L for each pesticide were prepared by dissolving accurately weighed 50 mg of each pesticide in a 50ml volumetric flask based on the pesticide solvent choice (heptachlor, cypermethrin, and deltamethrin, which were dissolved in methanol the rest studied pesticides were dissolve in acetone) and stored at 4°C. An intermediate solution of 100 mg/L was prepared by taking 1ml of stock solution and diluting it in 9 ml of the corresponding solvent. Working standard solutions were prepared at concentration ranges of 0.001 to 10 mg/L by serial dilution of the intermediate solution, and then the solution was stored in the refrigerator at 4°C. Calibration curves were drawn by running the working standard solutions for each pesticide.

### Instruments

Separation and quantification of the target pesticides were performed using Agilent Gas Chromatography equipped with an electron capture detector (GC-ECD), an auto sampler, a pump, and a column compartment model 7890A (Agilent Technologies, Singapore). An HP-5 capillary column (30 m, 0.25 mm inner diameter; and 0.25-mm film thickness) coated with 5% phenyl methyl siloxane (model 7890A) was also obtained from Agilent Technologies. This study also made use of a 15-mL centrifuge tube, analytical balance, a 5-mL syringe, and a centrifuge.

### Sample extraction procedures

The low-density-based dispersive liquid-liquid microextraction (LD-DLLME) method, which was earlier applied by [23], was used with minor modifications for the extraction of the target pesticide residues from water samples. Accordingly, a 5 mL water sample was taken into a 15 mL centrifuge tube, and then, a mixture of 100 μL N-hexane and 500 μL acetone, as extraction and disperser solvents, respectively, was rapidly injected using a 5 mL medical syringe. Subsequently, after the addition of 0.5 g of NaCl, the contents were shaken by hand until the salt was completely dissolved. Then the content was centrifuged for 5 minutes at 5000 rpm to facilitate phase separation. A 50 μL of the floating organic phase was carefully withdrawn via a syringe and then transferred into vials. Finally, 1 μL of the extract was injected into the gas chromatography system.

### Pesticide identification and quantification

Analyses of the pesticide residues were performed using GC-ECD. The GC-ECD was connected to a nitrogen gas generator of 99.99% purity, and used as a carrier and makeup gas. The instrument condition was set by the following conditions: ALS (auto liquid sampler) with 10 μL syringes was used and the injection volume was 1 μL. Split less injection mode was used with an inlet temperature of 250°C, a carrier nitrogen gas flow of 45 ml/min, and a pressure of 10.04 Psi. A column of 30 m x3.20 mm internal diameter and 0.25 μm film thickness was used following the oven temperature program with the initial temperature set at 80°C, ramp at a rate of 30°C/min to 180°C, then ramping at a rate of 3°C/min 205°C for 4 min holding time, and then ramping at a rate of 20°C/min 290°C for8 min holding time. The total run time was 27.92 min. The μECD detector temperature was 300°C using nitrogen as a makeup gas at a flow rate of 60 ml/min.

### Quality control

All glassware was washed with solvents before being used to remove any contaminants. The quantitative determination of pesticide residue in water was done based on the external standard method. Identification of the pesticides was undertaken by running specific concentrations for each pesticide and by looking at the retention time. And then, the linearity of the standard solutions was measured at five different concentrations of 10 mg/L, 1 mg/L, 0.1 mg/L 0.01 mg/L, and 0.001 mg/L from the mixture of all standards at their corresponding working concentration. Calibration curves were plotted by taking concentration against the peak area of the individual pesticides, and the linearity of the calibration curve was validated using a coefficient of determination (r^2^). Coefficients of regression (r^2^) were greater than 0.995 for all pesticides which indicate the linearity of standard curves. Limits of detection (LOD) and limit of quantification (LOQ), were determined as 3 and 10 times the signal-to-noise ratio, respectively (Table 1).

**Table 1:** Method validation results including recovery, LOD, and LOQ.

Pesticides	% Recovery	LOD (μg/l)	LOQ (μg/l)
p,p’-DDE	87	0.003	0.006
p,p’- DDD	84	0.02	0.01
o,p’-DDT	93	0.03	0.06
p,p’-DDT	92.2	0.002	0.002
ˠ-Chlordane	91	0.03	0.012
Lindane	111	0.12	0.113
Dimethachlor	89	0.04	0.014
Heptachlor	93	0.07	0.22
Cypermethrin	110.5	3	10
Deltamethrin	109.4	1	6

### Statistical analysis

An Excel sheet was used for the calculation of pesticide residues using the equation of the calibration curves. Mean and standard deviation was analyzed using SPSS ® V, 23. Shapiro-Wilk was used to check the normality of the data at *P*<0.05 was used. The one-way ANOVA test was used to examine the significant differences (Tukey’s test) (*p* < 0.05) for normally distributed data. In contrast, the Kruskal -Wallis nonparametric test was used for the data that were not normally distributed. *P-values< 0*.*05* were considered significant.

### Ethical consideration

Ethical clearance was obtained from the ethical review board of Jimma University Institute of Health before departure for sample collection and permission was obtained from Jimma Town drinking water treatment plant to take water samples. Moreover, informed verbal consent was received from all stakeholders.

## Results and discussion

The concentration of pesticides detected in the water samples from Jimma conventional water treatment plants in southwestern, Ethiopia at different processing, stages is illustrated in Table 2. The pesticides under study were DDT with its metabolites (p,p’-DDE, p,p’-DDD, p,p’-DDT, and o,p’-DDT), heptachlor, γ-Chlordane, lindane, dimethachlor, cypermethrin, and deltamethrin. From our findings, except for o´p-DDT, all the pesticides under study were detected at the intake point (the Gibe River, which is the source of water for the treatment plant) of the Jimma conventional treatment plant. This may be due to the current or historical illegal use of those banned pesticides or previously used pesticides by farmers, which could contaminate the source of water. Since these pesticides are highly persistent, the residue may exist for a long period of time in the water sources. Although organochlorine pesticides were widely used in the 1940s in large quantities, they were banned in the 1970sin developed countries, because of their high persistence in the environment and their harmful effects in human health.

**Table 2:** Mean concentration (μg/L) of pesticide residue in a different stage of the conventional water treatment process.

				Mean ± SD			
**Pesticides**	Raw Water	Aeration	Coagulation and Flocculation		Sedimentation	Filtration	Post- chlorination
**p´p-DDD**	70.83±55.49	69.27±15.29	78.05±49.18		107.98±45.15	148.81±85.18	62.54±6.86
**p´p-DDE**	0.31±0.11	ND	ND		ND	0.06±0.11	2.42±0.34**
**p´p-DDT**	158.10±58.38	ND*	ND*		18.24±0.54*	55.31±0.43*	75.92±22.80
**γ-Chlordane**	12.21±1.10	1.00±0.15	1.53±1.38		3.06±2.60	3.09±1.58	3.11±1.02
**Dimethachlor**	5510.10±723.41	342.83±73.73	367±208.7		6372.45±1249	1266.74±1047.4	149.50±127.99**
**Lindane**	1585.45±1337.5	809.53±146.8	356.17±194.7		223.18±99.3	663.11±405.89	135.47±101.27
**Heptachlor**	49.28±10.67	4.89±2.43	42.22±30.95		51.78±5.62	232.87±134.26	246.73±196.19
**Cypermethrin**	81.78±71.44	45.84±0.09	81.45±6.83		76.09±13.04	59.93±18.28	217.39±175.42
**Deltamethrin**	28.07±25.94	3.18±1.78	24.24±2.90		46.96±36.83	29.70±6.80	31.84±4.35

*represents ANOVA with Tukey post-hoc test results with P < 0.05 and

**represents Kruskal-Wallis test result with P < 0.05, ND = Not Detected.

Farmyard runoff, sewer overflows, and accidental spills [5] may contribute to the contamination of water in the study area. The mean concentration of p´p-DDD, p´p-DDE, and p´p-DDT in Gibe River was70.83 μg/L, 0.31 μg/L, and 158.1 μg/L, respectively. This is due to the fact that Jimma Zone in southwest Ethiopia was a malaria—endemic area, and these pesticides may be sprayed for public health purposes. The mean concentrations of cypermethrin and deltamethrin in the Gibe River were81.78 μg/L and 28.07 μg/L, respectively. These findings were lower relative to a study done in Nigeria by [24], the mean concentration of p´p-DDD, p´p-DDE, and p´p-DDT in River Water Benue in Vinikilang, Yola, Adamawa State, Nigeria were 2160 μg/L, 300 μg/L, and 3750 μg/L respectively. The concentrations of cypermethrin and deltamethrin in River Water Benue in Vinikilang, Yola, Adamawa State, Nigeria were 930 μg/L and 1140 μg/L, respectively. The mean concentration of lindane in the Gibe River of our study area was 1585.45 μg/L which is higher than river water, which is located in northern Greece detected at a concentration of 0.252 μg/L [25]. This may be due to residence area variation or the preferences of users or farmers toward different types of pesticides.

The mean concentration of pesticide residue in each processing stage of the conventional water treatment plant is investigated. The Kruskal-Wallis test result showed that there is a significant difference (p<0.05) in the mean concentration of p´p-DDE in raw water (0.31 μg/L) and post chlorination (2.42 μg/L). This may be due to a reaction between chlorine (post-chlorination) and chlorinated pesticides. The addition of chlorine can produce the transformation of parent compounds into their metabolites [4]. From one-way ANOVA test results, there is a significant difference (p< 0.05) between the mean concentration of raw water (158.1 μg/L), aeration (ND), coagulation (ND), sedimentation (18.24 μg/L), and filtration unit (55.31 μg/L) for p´p-DDT. This may be due to evaporation, photodecomposition, microorganisms, and the hydrophobicity characteristics of this pesticide contributing to the reduction of concentration by the above mentioned water treatment processes.

The mean concentration of the pesticides under study was analyzed from the Jimma water distribution system. Table 3 revealed that p´p-DDE and p´p-DDT were not detected in the Jiren and Abajifar reservoirs. The maximum mean concentrations of p´p-DDD, p´p-DDT, dimethachlor, cypermethrin, and lindane were detected at Gingo and Jimma University Medical Center reservoirs. But the minimum mean concentration of p´p-DDD, p´p-DDE, dimethachlor, lindane, heptachlor, cypermethrin, and deltamethrin was detected in Jiren and Abajifar reservoirs. This indicates a maximum mean concentration of pesticides is detected at Gingo and Jimma University Medical Center reservoirs; in contrast, a minimum mean concentration of pesticides is detected in Jiren and Abajifar reservoirs. The one-way ANOVA test results showed that there is a significant difference (p<0.05) in the mean concentration of dimethachlor between the Jimma University Medical Center reservoir and other reservoirs. These all might be due to the location variation of reservoirs, variation in the distribution line, and the difference in being near different agricultural activities and areas. Jiren and Abajifar reservoirs in Jimma, in south western Ethiopia, are located in a mountainous area and, there are no agricultural practices near these reservoirs, so the residues of pesticides became low. Since Ginjo and Jimma University medical center reservoirs are located in the lowlands, there might be farmyard runoff and sewer overflows that contribute to the increment of pesticide residue. A reservoir located at Jimma University Medical Center has no cover on top. This may contribute to the contamination of this reservoir with different pesticides coming from different activities, like farming.

**Table 3:** Pesticide residue concentrations (mean ± SD) in (μg/L) in Jimma town water distribution system.

Pesticides	TPR	GR	JUMCR	AR	JR	Tap
**p´p-DDD**	90.83 ± 58.85	128.55 ± 76.26	114.24 ± 39.02	79.0 ± 33.82	63.47 ± 40.66	95.91 ± 63.31
**p´p-DDE**	0.24 ± 0.01	0.43 ± 0.09	0.25 ± 0.05	0.1 ± 40	0.33	0.68 ± 0.65
**p´p-DDT**	3.88 ± 2.49	43.05 ± 28.22	62.14 ± 39.63	ND	ND	55.44 ± 43.22
**γ-chlordane**	2.75 ± 2.59	3.96 ± 2.62	4.63 ± 0.77	3.36 ± 0.5	9.78 ± 8.5	3.41 ± 2.58
**Dimethachlor**	223.63±171.9	294.87±157.55	1153.55±210.4*	85.47 ± 30.8	109.74 ± 1.53	359.56±249.27
**Lindane**	52.04 ± 23.74	180.91±120.35	181.04 ± 68.94	21.35 ± 1.6	42.98 ± 14.43	126.97±118.45
**Heptachlor**	73.62 ± 12.29	96.32 ± 77.67	83.77 ± 10.27	47.22 ± 2.4	50.02 ± 32.9	129.78 ± 91.58
**Cypermethrin**	98.81 ± 62.66	181.22 ± 58.14	119.51 ± 85.08	131.71 ± 92.35	80.28 ± 1.9	179.34±161.37
**Deltamethrin**	21.14 ± 10	33.14 ± 14.65	34.7 ± 9.16	22.54 ± 8.66	15.75 ± 2.63	37.78 ± 21.93

TPR: Treatment plant reservoir, GR: Ginjo reservoir, JUMCR: Jimma University Medical Center reservoir, AR: Abajifar reservoir, JR: Jiren reservoir, ND: not detected, SD: Standard deviation, * represents ANOVA with Tukey post-hoc test result with P < 0.05.

The mean concentration of p,p’-DDE, p,p’-DDD, p,p’-DDT, heptachlor, and lindane in the tap water of our study area was0.68 μg/L, 95.91 μg/L, 55.44 μg/L, 126.78 μg/L, and 126.78μg/L respectively. According to a study done in Afyonkarahisar, Turkey, the concentration of heptachlor was 0.041 μg/L, DDE 0.021 μg/L, and DDD 0.036 μg/L [26]. Lindane (0.03 μg/L), heptachlor (0.02 μg/L), and p´p-DDT(0.03 μg/L) were detected in Ghana’s drinking water [27]. The mean concentrations of cypermethrin and deltamethrin were, respectively, 179.34 μg/L and 37.78 μg/L in water samples collected from taps. Our finding was higher than the study done by Ghana [28], in which the mean concentration of cypermethrin was 0.03 μg/L and the mean concentration of deltamethrin was 0.05 μg/L. According to the World Health Organization (WHO) and the Netherlands government, the maximum residue level (MRL) value for total DDT, γ-Chlordane, and lindane is 2 μg/L, 0.2 μg/L and 2 μg/L respectively, and for Australia, the limit is 20 μg/L for total DDT. The MRL value for heptachlor is 0.03 μg/L, 0.3 μg/L, 0.4 μg/L, and 0.04 μg/L according to WHO, Australia, the United States of America(USA), and Netherlands guidelines, respectively [29]. Most pesticides that were detected in water samples from our study areas exceeded the maximum residue limits (MRLs) set by these organizations, except for p,p’-DDE. The exceedance of MRL confirms that the previous use or continuous illegal use of the pesticide in the study area as both parent and metabolites exceeds the legal limit (MRL).

The water treatment process may affect the level of pesticides. According to this study, during the aeration process, the concentration of the studied pesticides decreased. The percent reduction ranges from 2.21% (p´p-DDD) to 100% (p´p-DDE, p´p-DDT) by the aeration process (Table 4). This may be due to the evaporation and photodecomposition processes, which will contribute to the loss of pesticide residues. During coagulation and sedimentation, some pesticide residues became more concentrated, like dimethachlor, deltamethrin, heptachlor, and p´p-DDD. This may be due to a reaction with chlorine, which originates from the pre chlorination process. There is an addition of chlorine as pre chlorination with coagulant in the Jimma water treatment process. The addition of chlorine to the water treatment process can result in the reaction of pesticides, which will produce the transformation of parent compounds into metabolites [4].This may increase the concentration of pesticides during the chlorination process or result in a concentrated pesticide level.

**Table 4:** Percentage reduction of pesticide residue (%) through the conventional water treatment process.

Pesticides	Aeration	Coagulation & flocculation	Sedimentation	Filtration	Post-chlorination
**p´p-DDD**	2.21	-10.2	-52.45	-110.09	11.70
**p´p-DDE**	100	100	100	80.11	-688.28
**p´p-DDT**	100	100	88.46	65.02	51.98
**γ- Chlordane**	93.78	87.46	74.95	74.70	74.55
**Dimethachlor**	93.78	93.34	-15.65	77.01	97.29
**Lindane**	48.94	77.53	85.92	58.18	91.46
**Heptachlor**	90.07	14.33	-5.07	-372.54	-400.67
**Cypermethrin**	43.95	0.4	6.87	26.72	-165.82
**Deltamethrin**	88.67	13.62	-67.33	-5.81	-13.4 6

A negative sign indicated that the concentration of pesticides wasan increase during the treatment process relative to raw water.

During sedimentation, the percent reduction of pesticides ranged from 88.46% (p´p-DDT) to 6.87% (cypermethrin). Reduction in the concentration of pesticide during coagulation and sedimentation is due to the hydrophobic characteristics of organochlorine pesticides. These chemical pesticides are preferably bound to the particle phase in the aquatic system and then accumulate in the sediment or floc, which is supported by a study done by [24]; DDT metabolites were degraded by around 70% by the coagulation process, and 60% removal was obtained for heptachlor by peroxidation of chlorine [30]. During the filtration process, the percent reduction ranges from 80.11% (p´p-DDE) to 26.72% (cypermethrin). The reason for reducing the concentration of pesticide residues in the filtration processing stage might be due to microorganisms that are found in a biological layer on the sand surface known as the schmutzdecke, which have a role in the reduction of pesticide residues. Pesticides are used as a mainly microbial nutrient and ultimately decompose into some small molecules, such as CO_2_ and H_2_O. The progress is called an enzymatic reaction, which included the compound getting into the microorganism’s body in a certain way first, and then, through a series of physiological and biochemical reactions under the action of various enzymes, finally, the pesticide would be completely degraded or broken down into smaller molecular compounds that were non-toxic or less toxic [31,32]. Overall, p´p-DDD (11.7%), p´p-DDT (51.98%), γ Chlordane (74.55%), Dimethachlor (97.29%), and Lindane (91.46%) reduced their concentrations through the entire treatment process. Even if their concentration decreased, they would still exceed the MRL set by WHO, Australia, the USA, and the Netherlands. While the concentration of p´p-DDE was below the MRL, even if the concentration increased during the entire process, this might be due to the concentration of p´p-DDE at the intake point being very low. In another study in Iran, the concentration of chlorinated organic pesticides was decreased in most of the conventional water treatment processes. However, methoxychlor was concentrated from raw water in the sedimentation process [6].

The effect of the household water treatment process (boiling and solar disinfection) was investigated in this study. Boiling was not efficient for the removal of p´p-DDD, p´p-DDT, dimethachlor, lindane, heptachlor, cypermethrin, and deltamethrin. This might be due to the fact that the most studied pesticides are chlorinated and highly thermally resistant [33]. Only three pesticide concentrations were reduced by the boiling treatment process, namely; p´p-DDE, o´p-DDT, and γ-Chlordane. This may be due to the fact that these three pesticides have relatively lower boiling points than the other studied pesticides. O´p-DDT, p´p-DDE, and were removed 100%, 38.9%, and 27.13%, respectively, by the boiling process. The boiling points of DDT isomers and γ-Chlordane are 260°C and 175°C respectively. The rest of the pesticides have a boiling point range between 300°C and 350°C.

Using the SODIS treatment, the two pyrethroids (cypermethrin and deltamethrin) were removed by 54.45% and 15%, respectively. The one-way ANOVA test results showed that there is a significant difference (p<0.05) in the mean concentration of cypermethrin between the two treatment process. This may be due to the fact that photodegradation plays a significant role in the degradation of the product on leaf surfaces and surface waters, especially for pyrethroids (cypermethrin and deltamethrin) [34]. γ-Chlordane, heptachlor, lindane, p´p-DDD, and o´p-DDT were reduced by 44.81%, 26.2%, 14.45%, 5.38%, and 2.31%, respectively (Table 5).

**Table 5:** Mean concentration, percentage reduction, and PF of pesticides in the different household water treatment processes.

	Untreated	Boiled			SODIS		
Pesticides	Mean ± SD (μg/L)	Mean ± SD (μg/L)	Percent reduction (%)	PF	Mean ± SD (μg/L)	Percent reduction (%)	PF
**p´p-DDD**	668.65 ± 134.07	863.97± 113.53	-29.21	1.29	632.68 ± 451.69	5.38	0.95
**p´p-DDE**	14.23 ± 12.39	8.69 ± 3.27	38.90	0.61	24.34±12.03	-71.02	1.71
**p´p-DDT**	221.41 ± 156.76	312.32 ± 254	-41.06	1.41	347.35 ± 195.31	-56.88	1.57
**o´p-DDT**	89.69 ± 67.47	ND	100.00	0.00	87.62 ± 75.89	2.31	0.98
**γ-Chlordane**	64.46 ± 26.44	46.97 ± 34.25	27.13	0.73	35.58 ± 18.8	44.81	0.55
**Dimethachlor**	1096.22 ±331.13	1857 ± 593.35	-69.42	1.69	1290.41 ± 312.42	-17.71	1.18
**Lindane**	192.93 ± 38.85	457.77 ± 314.83	-137.27	2.37	165.05 ± 74.5	14.45	0.86
**Heptachlor**	418± 102.11	796.92 ± 660.01	-90.64	1.91	308.52 ± 161.7	26.20	0.74
**Cypermethrin**	12211 ± 8650.81	14428.2 ± 2371	-18.15	1.18	5563 ± 380*	54.45	0.46
**Deltamethrin**	200 ± 137	370 ± 120	-85	1.85	170 ± 40	15	0.85

*represents ANOVA with Tukey post-hoc test results with P < 0.05.

## Conclusions

Organochlorine pesticides (p,p’-DDE, p,p’-DDD, p,p’-DDT, heptachlor, γ-Chlordane, lindane, and dimethachlor) which were banned for agricultural use and two pyrethroids (cypermethrin and deltamethrin) that were detected in the Gibe River (intake point), in the distribution system, and in tap water (Tables 2 and 3). The presence of OCP in water samples indicates that there has been previous or recent illegal use of pesticides in the study area. Conventional water treatment processes reduce the concentration of p´p-DDT, p´p-DDD, γ-Chlordane, dimethachlor, and lindane to some extent (Table 4). However, their concentration still exceeds the MRL set by a different organization. This indicates there was no complete removal of these pesticides through the entire conventional water treatment process. Therefore, this may need additional intervention to protect the health of consumers. In addition, other alternative pesticides that are less harmful should be supplied to the farmers, and applying other advanced treatment mechanisms to reduce the level of pesticide residues in drinking water is important. The other results indicate that pesticides such as p,p’-DDE, p,p’-DDD, and o,p’-DDT, heptachlor, γ-Chlordane, lindane, cypermethrin, and deltamethrin were reduced through the household water treatment process, such as boiling or solar disinfection methods. However, p,p’-DDT and dimethachlor were not reduced during both household water treatment methods (SODIS and boiling) (Table 5). SODIS, which is not commonly applied by the public, should be recommended to assure the safety of the community. The other recommendation is that, other household water treatment methods, by changing different parameters should be applied. Generally, strict regulation is necessary, especially for those pesticides that are not removed by both treatment processes for keeping people safe and healthy.

This study would have some limitations because of does not include health risk assessment but the findings are sufficient to the effect of conventional and household water treatment technologies on the removal of pesticide residues in drinking water. For the future the authors plan to do detail risk assessment.

## Supporting information

S1 File Ethical approval. (PDF)
