# Supplementary material for: Effect of conventional and household water treatment technologies on the removal of pesticide residues in drinking water, Jimma town, Southwestern, Ethiopia
Source: PLoS One. 2023 Jul 19;18(7):e0288086. doi: 10.1371/journal.pone.0288086 (PMC10355405; doi:10.1371/journal.pone.0288086)
Supplement: S1 File — (PDF) [file pone.0288086.s001.pdf]

Jimma University Institute of Health

Institutional Review Board

Ref.No IHRPGD/131/21

Date: 11/5/2021

To: **Temima Jemal**

Subject: <u>Ethical Approval of Research Protocol</u>

The IRB of Institute of Health has reviewed your research project **"Impact of Household and Conventional Water Treatment Technologies on Pesticide Residues of Drinking Water in Jimma Town, Southwest Ethiopia"**

Thus, this is to notify that this research protocol as presented to the IRB meets the ethical and scientific standards outlined in national and international guidelines. Hence, we are pleased to inform you that your research protocol is ethically cleared.

We strongly recommend that any significant deviation from the methodological details indicated in the approved protocol must be communicated to the IRB before it has been implemented.

With Regards!

Million Tesfaye (MSc, PHD)
IRB chairperson
Tel: +251 913542906
E-mail: mtesfaye1@gmail.com

Jimma University Institute of Health Research & Innovation
